# AI-based online chat and the future of oncology care: a promising technology or a solution in search of a problem?

**DOI:** 10.3389/fonc.2023.1176617

**Published:** 2023-05-25

**Authors:** Joseph Kassab, Lewis Nasr, Georges Gebrael, Michel Chedid El Helou, Ludovic Saba, Elio Haroun, Joseph El Dahdah, Fadi Nasr

**Affiliations:** ^1^ Research Institute, Cleveland Clinic Foundation, Cleveland, OH, United States; ^2^ Department of Leukemia, The University of Texas MD Anderson Cancer Center, Houston, TX, United States; ^3^ Department of Hematology and Oncology, Saint-Joseph University of Beirut, Beirut, Lebanon; ^4^ Maroone Cancer Center, Cleveland Clinic Florida, Weston, FL, United States; ^5^ Division of Hematology and Oncology, State University of New York Upstate Medical University, Syracuse, NY, United States

**Keywords:** ChatGPT, chatbot, artificial intelligence - AI, oncology, assessment

## Introduction

The availability of medical information on the internet has transformed the dynamic of the patient-physician relationship. Patients are relying more and more on online resources to address their concerns. In November 2022, a dialogue-based artificial intelligence (AI) large language model (ChatGPT) was made accessible to the general public and has since gained millions of daily users (1, 2). Despite its capabilities in handling complex writing tasks (3), the use of chat-based AI in medicine is still in its early stages and is not intended for medical use yet. However, recent studies suggest a heightened potential for such technology to assist in patient education and provide supplementary information on various medical topics (4–10). We aimed to assess the ability of the AI model to provide accurate responses to evidence-based commonly asked patient-centered oncology questions.

## Methods

We convened a team of three oncologists from different institutions with extensive clinical and patient care experience to develop a set of thirty questions targeting common oncology topics. The questions focused on risk factors, preventive measures, diagnosis, treatment and side effects, and were designed in alignment with guideline-based topics, the oncologists’ clinical experience in both inpatient and outpatient settings and evidence based question prompt lists for patients seeing a medical or radiation oncologist (11, 12). The study was conducted in February 2023 using ChatGPT 3.5 (default model). The same questions were submitted to the online AI model three times to ensure consistency. Each set of 30 answers was assigned to 1 of the 3 oncologists. Each oncologist qualitatively graded each answer based on its relevance to current published preventive and management guidelines (13, 14) and their clinical expertise. They classified each response as either ‘accurate’, ‘inaccurate’, or ‘harmful’. An answer was considered accurate if – according to the oncologist – it contained essential and appropriate information, inaccurate if it provided false or incomplete information, and harmful if it included potentially harmful information for the patient. The oncologists’ assessments were then reviewed, and their evaluations combined. Evidently, to limit subjectivity and selection bias, an answer was deemed ‘accurate’ if all three oncologists judged it to be accurate, ‘inaccurate’ if at least one oncologist deemed it inaccurate, and ‘harmful’ if at least one oncologist deemed it harmful.

## Results

AI model responses were considered “accurate” in 26 of 30 questions (86%). 4 responses (14%) were considered “inaccurate”, and none were considered “harmful” (**Table 1**). For example, when asked about hair loss as a side effect of immunotherapy, the AI model did not provide an adequate response and gave false but non-harmful information. Nonetheless, when asked about preventive strategies and screening recommendations for various types of cancer, such as breast lung cancer and colorectal cancer, the AI was in line with current recommendations. The average response time was approximately 0.18 seconds per word.

**Table 1:** Assessment of Oncology Topics Recommendations by Experienced Oncologists using an Online Chat-Based Artificial Intelligence Model.

Question	Accuracy graded by oncologists**
Risk factors	
**Does diet affect cancer risk?**	Accurate
**If I stop smoking will it reduce my risk of getting cancer?**	Accurate
**Will I get breast cancer if I have a family history?**	Accurate
**Is obesity linked to cancer?**	Accurate
**Do oral contraceptive pills increase my risk of cancer?**	Accurate
**Does getting a CT scan increase the risk of getting cancer?**	Inaccurate
**Is there a vaccine for cancer?**	Inaccurate
**Does alcohol consumption increase cancer risk?**	Accurate
**Does physical activity reduce cancer risk?**	Accurate
**My city has a high pollution index, am I at higher risk of getting cancer?**	Accurate
Preventive medicine & Diagnosis	
**Should I screen for prostate cancer?**	Accurate
**How can I screen for breast cancer?**	Accurate
**Is there a way to prevent colorectal cancer?**	Accurate
**Can I screen for lung cancer?**	Accurate
**Can I prevent cervical cancer?**	Accurate
**What does cancer stage mean?**	Accurate
**Can I do a genetic test to know if I will get cancer?**	Accurate
**What is the difference between CT and PET scan?**	Accurate
**What is a biopsy and why do I need it?**	Inaccurate
**Can cancer be detected on regular blood tests?**	Accurate
Treatment and Side effects	
**What is immunotherapy?**	Accurate
**Will I lose my hair after immunotherapy?**	Inaccurate
**I am starting chemotherapy tomorrow what are the side effects?**	Accurate
**How can I prevent hair loss after chemotherapy?**	Accurate
**Will cancer treatment affect my fertility?**	Accurate
**How can I reduce nausea after chemotherapy?**	Accurate
**Can I go out in public after chemotherapy?**	Accurate
**Can herbal treatments replace chemotherapy?**	Accurate
**What is a clinical trial?**	Accurate
I feel down since starting chemotherapy. What do I do? *	Accurate

*Answer sample: It is common to experience emotional changes during and after chemotherapy treatment. Some people may experience feelings of sadness, anxiety, or depression. This can be due to the physical and emotional stress of cancer and its treatment, as well as the changes in hormone levels and other physical factors. If you are feeling down, it is important to talk to your healthcare team. They may be able to suggest strategies for managing your emotions and provide support and resources. Some strategies that may be helpful include: 1) Exercise: Physical activity can help boost mood and reduce stress. 2) Eating a healthy diet: A balanced diet that includes plenty of fruits, vegetables, and whole grains can help improve mood and energy levels. 3) Talking to someone: Talking to a friend, family member, or mental health professional can help you process your feelings and provide emotional support. 4) Mindfulness practices: Techniques such as meditation, deep breathing, and yoga can help reduce stress and anxiety. 5) Participating in activities you enjoy: Doing things that bring you pleasure, and joy can help improve your mood and reduce feelings of depression. 6) It is also important to know that there are medications and therapies available to treat depression, and your healthcare team can help you find the right treatment for you. Don’t hesitate to reach out for support and help if you are feeling down.

** An answer was deemed ‘accurate’ if all three oncologists judged it to be accurate, ‘inaccurate’ if at least one oncologist deemed it inaccurate, and ‘harmful’ if at least one oncologist deemed it harmful. Interviewer agreement of inaccurate responses was not assessed.

## Discussion

We reported that a popular online chat-based AI model provided mostly accurate responses to many oncology-related questions as evaluated by experienced oncologists. These findings suggest the potential of interactive AI in assisting in patient education. This could be particularly beneficial for oncology patients who may have complex and diverse questions regarding their condition. For example, the use of AI in this setting could potentially alleviate the need for patients to search for answers across numerous websites, which can often be overwhelming, time-consuming, and provide conflicting information. Nonetheless, the added value of such AI-based online chat remains heavily debated. With 14% of answers being inaccurate, the risk of receiving incorrect recommendations is significant. While future medically validated AI-based online chat could eventually provide patients with accurate supplementary information and resources, it is important for patients to understand that these technologies cannot replace the essential role of patient-physician interaction in making informed decisions about treatment options and managing care.

This study has several limitations. Firstly, it is important to note that the currently used AI model used is not intended for actual medical purposes. Secondly, oncology being a complex field, it cannot be fully covered by the limited set of questions used in this study. Thirdly, the accuracy and reliability of AI are susceptible to limitations and biases in the training data provided by the developers, which may result in incorrect or outdated information being provided. Fourthly, the evaluation of the responses was qualitative and conducted by a group of oncologists who may not represent the diverse views and opinions of healthcare professionals across different settings. Fifthly, inter-reviewer agreement and heterogeneity between the set of 3 AI responses was not assessed in detail. Finally, the AI responses did not include references to supporting evidence.

Future research should focus on developing a more standardized system for grading responses and assessing accuracy through a clear comparative analysis between physicians’ answers and AI-generated answers, as well as evaluating the usefulness of such tools in supporting physician practice.

## Author contributions

JK: Drafted the manuscript. All authors contributed to the editing and writing. All authors have read and approved the manuscript. All authors contributed to the article and approved the submitted version.
